# The 2016-2017 Chikungunya Outbreak in Karachi

**DOI:** 10.1371/currents.outbreaks.7257f6b05d8c18cf9e6eb222248be79f

**Published:** 2018-08-07

**Authors:** Darakhshan Guhar, Nadia Jamil, Shoukat Jahan Talpur, Gulzar Ahmed Channa, Maliha Wajeeh, Muhammad Zohaib Khan, Saifullah Khan

**Affiliations:** Centres of Excellence in Science and Applied Technology, Karachi, Pakistan; Centre for Molecular Biology, Centres of Excellence in Sciencechnology, Karachi, Pakistan.e and Applied T; Centres of Excellence in Science and Applied Technology, Karachi, Pakistan; Centres of Excellence in Science and Applied Technology, Karachi, Pakistan; Centres of Excellence in Science and Applied Technology, Karachi, Pakistan; Centres of Excellence in Science and Applied Technology, Karachi, Pakistan; Centres of Excellence in Science and Applied Technology, Karachi, Pakistan

## Abstract

Introduction: Chikungunya is an incipient disease, caused by Chikungunya virus (CHKV) that belongs to genus alphavirus of the family Togaviridae.

Materials and Methods: In this study, during an outbreak of CHKV in Dec 2016 in Karachi, Pakistan, samples were collected from patients presenting with fever, tiredness and pain in muscles and joints. Total 126 sera were tested for the presence of Chikungunya infection through ELISA and Real-time Reverse Transcriptase PCR assay.

Results and Discussion: This study showed that approx 79.4% samples were positive for CHKV. To our knowledge, this is the first reported outbreak from last decades in which the presence of CHKV is confirmed in Karachi while affecting such large no. of individuals.. Conclusion: CHKV diagnosis should be considered by the scientists and clinicians as a differential diagnosis in febrile patients, and appropriate control strategies must be adopted for its surveillance.

## **Introduction**

Chikungunya is a fever causing contagion which is transmitted to humans through the bite of CHKV harboring mosquitoes A. aegypti and A. Albopictus. The name “chikungunya” is derived from a word of the Kimakonde language which means “to become contorted”, and describes the stooped appearance of sufferers with joint pain (arthralgia). CHKV generally causes mild illness but could lead to severe life-threatening complications. The disease is characterized by an acute illness with fever, chills, headache, nausea, vomiting, joint pain, low back pain, and skin rash. CHKV causes arthralgia, which may persist for months 1^, ^2^, ^3 . The incubation period of CHKV ranges from 2–10 days, with statutory symptoms lasting up to 7 days. The symptoms usually resolve within days to a few weeks; but in severe cases, these symptoms may last for months. Currently, chikungunya fever has affected more than 50 countries. The global distribution of A. aegypti is expanding owing to global travel and trade, and so does the virus. It has become a public health problem in Asia, Africa, Europe and America. In 1983 chikungunya existence was reported in rodents in Pakistan. Until 2007 Pakistan was not included in CDC list. In 2008 Pakistan appeared in the list and cases were first reported in 2011 when dengue cases were atpeak in the country. During Dengue fever outbreak in 2011 a few patients with chikungunya were reported in Lahore 4 . Current outbreak of 2016-17 was initially termed as Mysterious disease 5 associated with the warm climate and inferior sanitary state of the city 4 . According to a local report total suspected cases between Dec 19, 2016, and Feb 22, 2017 were 818 6 , and according to WHO a total of 1018 suspected cases of chikungunya were reported between 19 December 2016 to 30 March 2017, in various districts of Karachi. No deaths have been reported so far 7 . These cases were clinically evaluated at various hospitals and labs in Karachi and Islamabad. At NIH, Islamabad 121 samples out of 157 samples were confirmed for CHKV infection via laboratory diagnosis 7 . In this study, total 126 serum samples from febrile illness patients were evaluated at CESAT to confirm an outbreak of CHKV infection in Karachi for the first time.

## **Materials and Methods**

During December 2016, clinicians in Karachi (Pakistan) observed more than 1000 patients (in a single day) presenting with fever, body rash, swollen joints and joint pain in three large hospitals of Karachi at the area of Saudabad, Malir and its surrounding.

**Sample Collection: **Total 126 blood samples were mainly collected from suspected patients admitted in emergency of Saudabad Sindh Govt Hospital, Karachi. These patients were presented with fever, chills, body pain, headache, arthralgia and anorexia. Most of the patients had stooped figure due to pain. Blood samples were collected in gel tubes and EDTA tubes for ELISA and PCR respectively.

**Demographics and Travel History:** Total 68 male and 58 female patients were included in this study. None of the patients had history of traveling abroad prior to infection. Patients were mostly from Malir Area of Karachi which is a mosquito endemic area, with low hygienic conditions.

**Selection Criteria:** The inclusion criteria for the patient were high grade fever, severe joint pain, stiff hands and stooped figure.

**ELISA: **Serum was separated from blood samples and all the serum samples were screened for the presence of Immunoglobulin M (IgM) antibodies against CHKV using a microtiter plate ELISA assay. The ELISA test was performed according to the kit manufacture’s protocol and interpreted either positive or negative on the basis of absorbance with respect to cutoff values.

**PCR:** In the present study, RNA samples were also analyzed for molecular detection by using Real-time Reverse Transcriptase PCR. Chikungunya infections results in high levels of viremia, which typically last for 4–6 days after the onset of illness. During the acute phase of infection viral RNA can be easily detected by reverse transcriptase-polymerase chain reaction (RT-PCR) in serum samples obtained from patients. RT-PCR can therefore easily be done within the first 7 days on an acute-phase specimen to confirm chikungunya virus infection. RNA was extracted from 400μl of serum samples. One-step Real time PCR assay was carried out using Primer pair and Probes specific to CHKV. In singleplex reaction mixture 11μl of RNA template was added with 14 μl of PCR Master mix (Invitrogen), to make 25 μl total.

**Ethics Statement: **Samples were collected after receiving approval from the local hospital Ethics Committee. Obtained written informed consent was received from the participants.

## Results & Discussion

The CHKV and DENV vectors i.e. Ae. Aegypti and Ae. Albopictus, already exist and thrive in Pakistan. Therefore, both Chikungunya and Dengue virus have opportunity of infecting the community on large scale. Furthermore, the initial signs and symptoms of both Dengue and Chikungunya are quite similar, which may lead to difficulties in making an appropriate provisional diagnosis. Laboratory diagnosis by ELISA and PCR both plays a vital role for differential diagnosis between Dengue and Chikungunya.

Out of total of 126 suspected serum, thirty eight (38) samples were positive for anti-Chikungunya IgM by ELISA, which suggest that these patients were in post viremic or convalescent phase. Three (03) patients were found positive for anti-Chikungunya IgG by ELISA alone which suggest that these patients were in post viremic or late stage after infection. Forty nine (49) samples were found positive by Real-Time Reverse Transcriptase PCR assay alone, which suggest that these patients were in viremic or acute phase. However, eighteen (18) samples were found positive by ELISA and PCR both, which suggest that these patients were in transitional phase.

This study revealed that overall 100 (79.4%) patients were found affected out of 126 suspected patients during this outbreak. This is a huge number of affected populace in a single city at a time.

## Conclusion

This study confirmed that the current outbreak in Karachi during Dec 2016-Feb 2017 is of Chikungunya Virus and may appeared in more frequent outbreaks of CHKV in near future as of dengue in Pakistan.

We recommend surveillance for CHKV, its vectors and preparedness to prevent future outbreaks of CHKV infection in Pakistan.

## Corresponding Author

Dr. Nadia Jamil is the corresponding author for this article. Email: diaraj@hotmail.com

## Data Availability Statement

The raw data required to reproduce these findings are available to download from the following Figshare link: https://figshare.com/articles/Probes_and_Primers_details/6917054.

Example photographs can be found via the following Figshare DOIs: 10.6084/m9.figshare.6819419; 10.6084/m9.figshare.6819431; 10.6084/m9.figshare.6819434; 10.6084/m9.figshare.6819437; 10.6084/m9.figshare.6819440; 10.6084/m9.figshare.6819443.

## Competing Interests Statement

There are no competing interests.

## 
